# Profile of Immunoglobulin G *N*-Glycome in COVID-19 Patients: A Case-Control Study

**DOI:** 10.3389/fimmu.2021.748566

**Published:** 2021-09-23

**Authors:** Haifeng Hou, Huan Yang, Pengcheng Liu, Changwu Huang, Meng Wang, Yuejin Li, Mingsong Zhu, Jing Wang, Yuan Xu, Youxin Wang, Qingwei Ma, Dong Li, Pu Liao, Wei Wang

**Affiliations:** ^1^ School of Public Health, Shandong First Medical University & Shandong Academy of Medical Sciences, Tai’an, China; ^2^ School of Clinical Medicine, Southwest Medical University, Luzhou, China; ^3^ Department of Clinical Laboratory, Chongqing General Hospital, Chongqing, China; ^4^ Department of Clinical Laboratory, The Fifth People’s Hospital of Chongqing & Chongqing Renji Hospital, University of Chinese Academy of Sciences, Chongqing, China; ^5^ Department of Clinical Laboratory, Chongqing Public Health Medical Center, Chongqing, China; ^6^ Beijing Key Laboratory of Clinical Epidemiology, School of Public Health, Capital Medical University, Beijing, China; ^7^ Testing Center, Bioyong Technologics, Inc., Beijing, China; ^8^ Centre for Precision Health, School of Medical and Health Sciences, Edith Cowan University, Perth, WA, Australia

**Keywords:** COVID-19, glycosylation, IgG, SARS-CoV-2, case-control study

## Abstract

Coronavirus disease 2019 (COVID-19) remains a major health challenge globally. Previous studies have suggested that changes in the glycosylation of IgG are closely associated with the severity of COVID-19. This study aimed to compare the profiles of IgG *N*-glycome between COVID-19 patients and healthy controls. A case-control study was conducted, in which 104 COVID-19 patients and 104 age- and sex-matched healthy individuals were recruited. Serum IgG *N*-glycome composition was analyzed by hydrophilic interaction liquid chromatography with the ultra-high-performance liquid chromatography (HILIC-UPLC) approach. COVID-19 patients have a decreased level of IgG fucosylation, which upregulates antibody-dependent cell cytotoxicity (ADCC) in acute immune responses. In severe cases, a low level of IgG sialylation contributes to the ADCC-regulated enhancement of inflammatory cytokines. The decreases in sialylation and galactosylation play a role in COVID-19 pathogenesis *via* the activation of the lectin-initiated alternative complement pathway. IgG *N*-glycosylation underlines the complex clinical phenotypes of SARS-CoV-2 infection.

## Introduction

Coronavirus disease 2019 (COVID-19), caused by severe acute respiratory syndrome coronavirus 2 (SARS-CoV-2), remains a global health challenge. COVID-19 has resulted in more than 194.0 million infections and 4.1 million deaths as of July 26, 2021 (1). SARS-CoV-2 is a positive-sense single-stranded RNA virus with four structural proteins: small envelope (E), matrix (M), nucleocapsid (N), and spike (S) (2). The transmembrane S protein is extensively glycosylated with a total of 22 *N*-linked glycan sequons per protomer which mediate infectivity and immune escape (3, 4). While understanding that the glycan structures of SARS-CoV-2 potentially assist vaccines design, antibody therapeutics, screening of small-molecule drugs and their targets (5–7), and recognition of the human immunoglobulin G (IgG) glycome hold the similar relevance to investigate the susceptibility and complicated clinical phenotypes of the SARS-CoV-2 infection.

As a set of glycoproteins, IgG is attached with two *N*-linked glycans at asparagine 297 sites of the fragment crystallizable (Fc) segments in heavy chains (8, 9). These glycans modulate the inflammatory properties of IgG by changing the affinity for specific receptors and lectins (10), which are involved in the development of inflammatory diseases (11–16). Progression of dengue virus (DENV) infection attributed to antibody-dependent enhancement (ADE) is proved to be regulated by specific IgG glycosylation (17). However, there is limited knowledge on IgG glycosylation that is associated with SARS-CoV-2 infection. Recently, the levels of bisecting *N*-acetylglucosamine (GlcNAc) and galactosylation of IgG were reported to be negatively correlated to the severity of the European COVID-19 patients (18). Meanwhile, IgG afucosylation was observed to be positively correlated with the severity of COVID-19 (19). Moreover, a study showed that IgG against SARS-CoV-2 S protein is characterized by afucosylation, whereas IgG against N protein has a higher level of fucosylation (20). These findings support the notion that changes in IgG glycome composition are closely related to the loss of the immunosuppressive function and contribute to the immune-mediated pathologies of SARS-CoV-2 infection (18). We aimed to profile the IgG *N*-glycome in COVID-19 patients by an age- and sex-matched case-control study.

## Materials and Methods

### Study Participants

We randomly enrolled 104 COVID-19 patients who were hospitalized in Chongqing Public Health Medical Center from January 24 to May 6, 2020. The inclusion criteria are as follows: (1) diagnosed in accordance with the eighth edition of the Diagnosis and Treatment Plan for COVID-19 issued by the National Health Commission of China both male and female patients with COVID-19; (2) definitive diagnosis using polymerase chain reaction (PCR) test; (3) did not receive vaccines in the preceding 6 months; and (4) not enrolled in other clinical trials. The exclusion criteria are as follows: (1) patients with other viral diseases; (2) patients who have been undergoing chemotherapy for the past month; (3) patients with severe diseases, such as coronary heart disease, stroke, and cancers; and (4) patients with autoimmune diseases. All patients were recruited at the first week of hospitalization. As controls, 104 age- and sex-matched healthy participants were enrolled from a local community-based cohort. The Ethical Committee of Chongqing General Hospital approved this study (No. S2020-021-01). Informed consent was obtained from each study participant.

### Analysis of Immunoglobulin G Glycans

Isolation of IgG and release of *N*-glycans were performed as described previously (21, 22). Briefly, the frozen sera were thawed, and were centrifugated at 80 g for 10 min. Dilute the samples with 1× phosphate-buffered saline (PBS, pH = 7.4) by 1:7 (v/v). The samples were then transferred to the protein G monolithic plate, for IgG binding and cleaning, followed by PBS washing as previously reported. IgG samples were eluted with 1 ml of 0.1 M formic acid and filtered into the collection plate by a vacuum pump. And, 170 µl of 1 M ammonium bicarbonate was added to each plate with shock blending.

Isolated IgG samples were denaturized with 20 µl of 2% sodium dodecyl sulfonate (v/v) in a 65°C oven for 10 min. Then, 10 µl of 4% IGEPAL was added and incubated in a shaking incubator for 5 min. After the samples were regulated by (30 ul) 0.1 mol/L NaOH and 20 µl of 5 × PBS to a pH of 8.0 with shock blending, then 4 µl of PNGase F enzyme were added. And the samples were incubated in a 37°C water bath for *N*-Glycan release.

The released *N*-glycans were labeled with 35 µl of 2-aminobenzamide(2-AB) mixed solution reagent, and transferred into an oscillator for about 10 min, then transferred into an oven at 65°C for 3 h. The 2-AB labeled glycans were purified by 100% acetonitrile, and eluted with 100 µl of ultra-pure water. The 2-AB labeled glycans were transferred into an oven to dry at 60°C for 3.5 h, and then saved at -80°C until further measurement.

*N*-glycan samples were dissolved with a mixture of 100% acetonitrile and ultra-pure water at a 2:1 ratio (v/v). Then, it was centrifuged at 134 g for 5 min and 20 µl of the labeled N-glycans was loaded into the UPLC instruments. The glycans were analyzed with hydrophilic interaction liquid chromatography (HILIC) on a UPLC instrument (Walters Corporation, Milford, MA) (22, 23). A total of 24 chromatographic glycan peaks (GPs 1–24) were identified as initial glycans. Each glycan was quantified as relative values of the GP to total GPs. The glycan structure in each peak was matched as previously reported (22). We further calculated the derived glycans using the measurement data of the initial GPs, which consist of sialylation trait, bisecting GlcNAc trait, galactosylation trait, and fucosylation trait (11). Statistical analyses were performed on each glycan, and also on the summary features of the IgG glycome composition, i.e., G1: glycans with one galactose, G2: glycans with two galactoses, G0: glycans without galactose, S: sialic acid, F: fucose, and B: bisecting GlcNAc.

### Statistical Analysis

Normal distribution of all analysis results was checked using the Kolmogorov-Smirnov test. Continuous variables underlying the abnormal distribution were represented as the medians (interquartile ranges). The difference of continuous variables between two groups was tested using the Wilcoxon rank-sum test. Statistical analyses were carried out with the SPSS software, version 25.0 (IBM, New York, USA). All reported P-values were two-sided, and a P < 0.05 was considered statistically significant.

## Results

### Characteristics of Study Participants

In this case-control study, we recruited 104 COVID-19 patients (50 men/54 women, mean age 44.81 years) from Chongqing Public Health Medical Center, Chongqing, China. Simultaneously, 104 age- and sex-matched healthy participants were enrolled from a local community-based cohort. Of the 104 COVID-19 patients, 18 were severe cases (with respiratory rate ≥ 30 times/min, oxygen saturation ≤ 93% on ambient air), and 86 were mild cases. The characteristics of the study participants are shown in **Table 1**.

**Table 1:** Characteristics of study participants.

Variables	COVID-19 patients(n = 104)	Controls(n = 104)	*P*
Sex (men/women)	50/54	50/54	–
Age	44.81 ± 13.96	44.62 ± 11.629	0.918
WBC	5.31 (4.23–6.73)	6.02 (4.89–7.11)	0.005
RBC	4.35 (3.95–4.65)	4.67 (4.25–5.04)	<0.001
PLT	201.00 (266.00–272.00)	262.00 (222.00–296.00)	<0.001
ALT	22.00 (17.00–39.00)	24.00 (18.00–33.00)	0.357
AST	20.00 (17.00–27.00)	21.00 (18.00–26.00)	0.518
Cr	64.55 (57.60–74.18)	66.00 (57.00–74.00)	0.921
UA	307.77 ± 86.06	305.22 ± 74.82	0.588
FBG	5.72 (5.32–7.06)	5.67 (5.32–6.21)	0.118
Hypertension	6 (5.77%)	0	–
Hyperlipidemia	2 (1.92%)	0	–
Hyperuricemia	1 (0.96%)	0	–
Diabetes	11 (10.58%)	0	–

ALT, alanine aminotransferase; AST, aspartate aminotransferase; Cr, serum creatinine; FBG, fasting blood glucose; PLT, platelet; RBC, red blood cell; UA, serum uric acid; WBC, white blood cell.

### The Immunoglobulin G Glycome Composition in Participants

A total of 24 initial glycan peaks (GP1–GP24) were obtained from all the chromatograms of UPLC. As shown in **Table S1**, 10 GPs (GP1, GP3, GP5, GP11, GP13, GP17, GP20, GP21, GP22, and GP23) were significantly higher in COVID-19 patients than that of the controls (**Table S2**), whereas another 12 GPs (GP2, GP4, GP6, GP8, GP9, GP10, GP12, GP14, GP16, GP18, GP19, and GP24) were lower in COVID-19 patients. We further calculated the derived glycans traits using the measurement data of the initial GPs, which consist of sialylation, bisecting GlcNAc, galactosylation trait, and fucosylation traits (11). As listed in **Table S2**, 39 of 54 derived glycans were differential between the COVID-19 patients and the controls.

### Fucosylation

**Figure 1:**
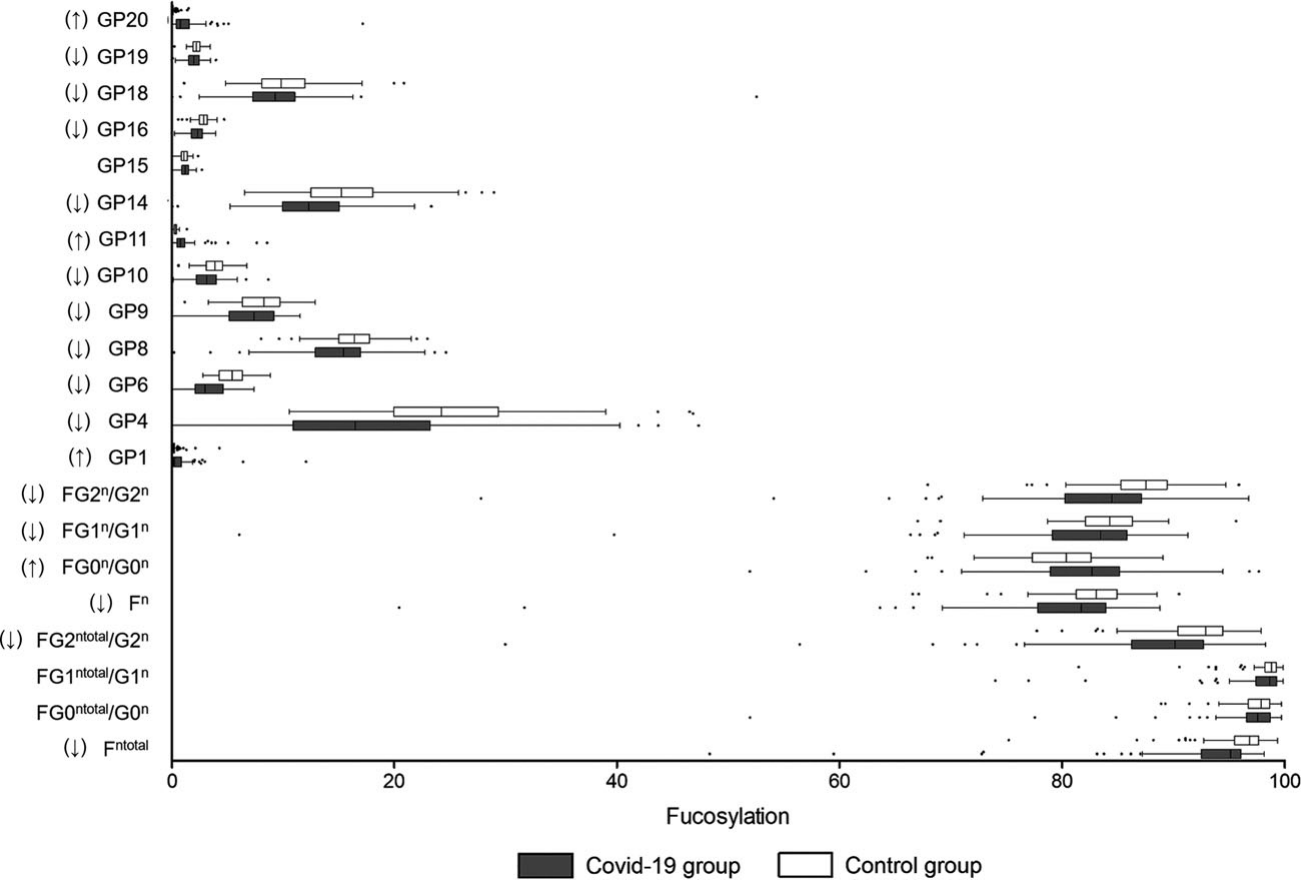
Fucosylation on IgG in COVID-19 patients and the healthy controls. B, bisecting N-acetylglucosamine (GlcNAc); F, fucose; G, galactose; GP, glycan peak; S, Sialic acid; ↑, the glycan in COVID-19 group is significantly higher than that in the controls; ↓, the glycan in COVID-19 group is significantly lower than that in the controls.

Afucosylation upregulates the capability of IgG to trigger antibody-dependent cell cytotoxicity (ADCC) and leads to the enhancement of inflammatory cytokines produced by monocytes. An increase in IgG afucosylation has been observed among severe COVID-19 patients in earlier studies involving Caucasian populations (18, 20). As shown in **Figure 1**, 14 fucosylated glycans (GP4, GP6, GP8, GP9, GP10, GP14, GP16, GP18, GP19, F^n total^, FG2^n total^/G2^n^, F^n^, FG1^n^/G1^n^, and FG2^n^/G2^n^) were significantly lower in the COVID-19 group when compared to the controls. The percentage of glycans with fucose was 82.54% in the COVID-19 cases, which was significantly lower than that in the control group (95.64%) (**Table 2**).

**Table 2:** Relative abundance (%) of the main IgG glycome features in COVID-19 patients and the healthy controls.

Summary glycans	COVID-19 patients (n = 104)	Controls (n = 104)	*Z*	*P*
Fucosylation	82.54 (72.36–87.21)	95.64 (94.12–96.69)	10.874	<0.001
Bisecting GlcNAc	14.30 (12.57–15.66)	15.39 (13.48–17.08)	2.398	0.016
Galactosylation	76.13 (67.14–83.02)	69.21 (63.41–72.93)	4.668	<0.001
Sialylation	29.68 (21.75–39.29)	20.60 (17.48–23.82)	6.296	<0.001

GlcNAc, N-acetylglucosamine.

However, the levels of fucosylated IgG in the severe COVID-19 cases (87.94%) were significantly higher than those in the mild cases (85.54%), which might be attributed to the heterogeneity of age between severe and mild cases (**Table 3**). Furthermore, we analyzed the core fucosylation of IgG in the severe COVID-19 cases compared to the mild patients. As listed in **Table S3**, no differences in core fucosylated moieties were identified between the severe and mild cases.

**Table 3:** Relative abundance (%) of the main IgG glycome features in severe and mild COVID-19 patients.

Summary glycans	Severe patients (n = 18)	Mild patients (n = 86)	*Z*	*P*
Fucosylation	87.94 (82.05–92.63)	85.54 (82.22–90.22)	3.342	0.001
Bisecting GlcNAc	13.22 (11.81–15.68)	15.38 (13.48–17.08)	1.413	0.158
Galactosylation	58.32 (53.57–62.47)	74.07 (64.54–75.71)	4.734	<0.001
Sialylation	18.93 (8.73–24.81)	26.09 (17.79–32.33)	3.746	<0.001

GlcNAc, N-acetylglucosamine.

### Sialylation

**Figure 2:**
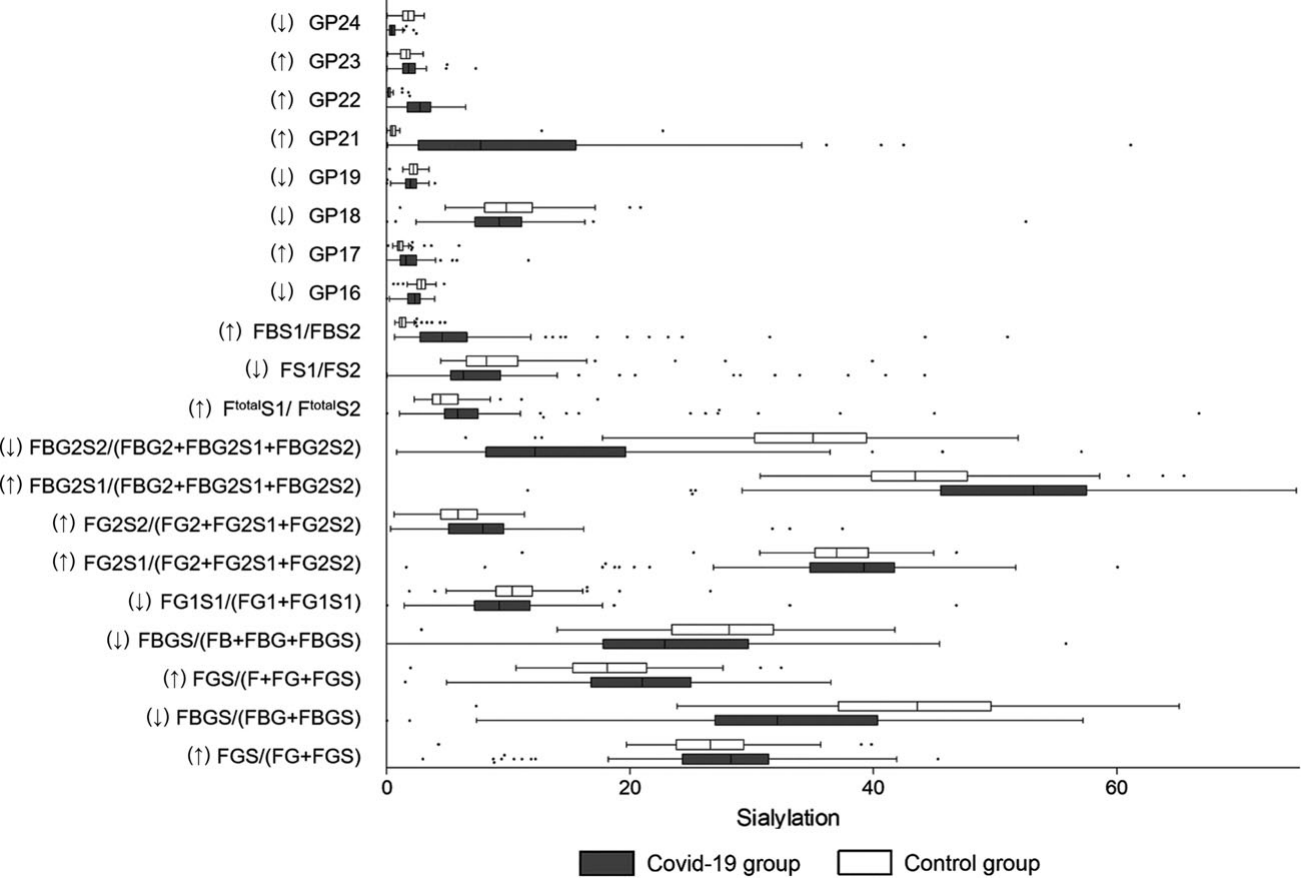
Sialylation on IgG in COVID-19 patients and the healthy controls. B, bisecting N-acetylglucosamine (GlcNAc); F, fucose; G, galactose; GP, glycan peak; S, Sialic acid; ↑, the glycan in COVID-19 group is significantly higher than that in the controls; ↓, the glycan in COVID-19 group is significantly lower than that in the controls.

IgG sialylation, also known as N-acetylneuraminic acid (Neu5Ac)-linked IgG, regulates pro-inflammation and anti-inflammation balance. The decrease in IgG sialylation upregulates the antibody-dependent cell cytotoxicity (ADCC) pathway and is linked to activation of the lectin-initiated alternative complement pathway (24). In this study, 11 sialylated glycans (GP17, GP21, GP22, GP23, FGS/(FG+FGS), FGS/(F+FG+FGS), FG2S1/(FG2+FG2S1+FG2S2), FG2S2/(FG2+FG2S1+FG2S2), FBG2S1/(FBG2+FBG2S1+FBG2S2), F^total^S1/F^total^S2, and FBS1/FBS2) were higher in the COVID-19 patients (**Figure 2**). In total, a higher abundance of sialylated glycans was identified in the COVID-19 group (29.68%) than that in the controls (20.60%) (**Table 2**). Meanwhile, a lower level of total sialylated glycans was observed in the severe patients (18.93%) than that in the mild cases (26.09%) (**Table 3**).

### Bisecting GlcNAc

**Figure 3:**
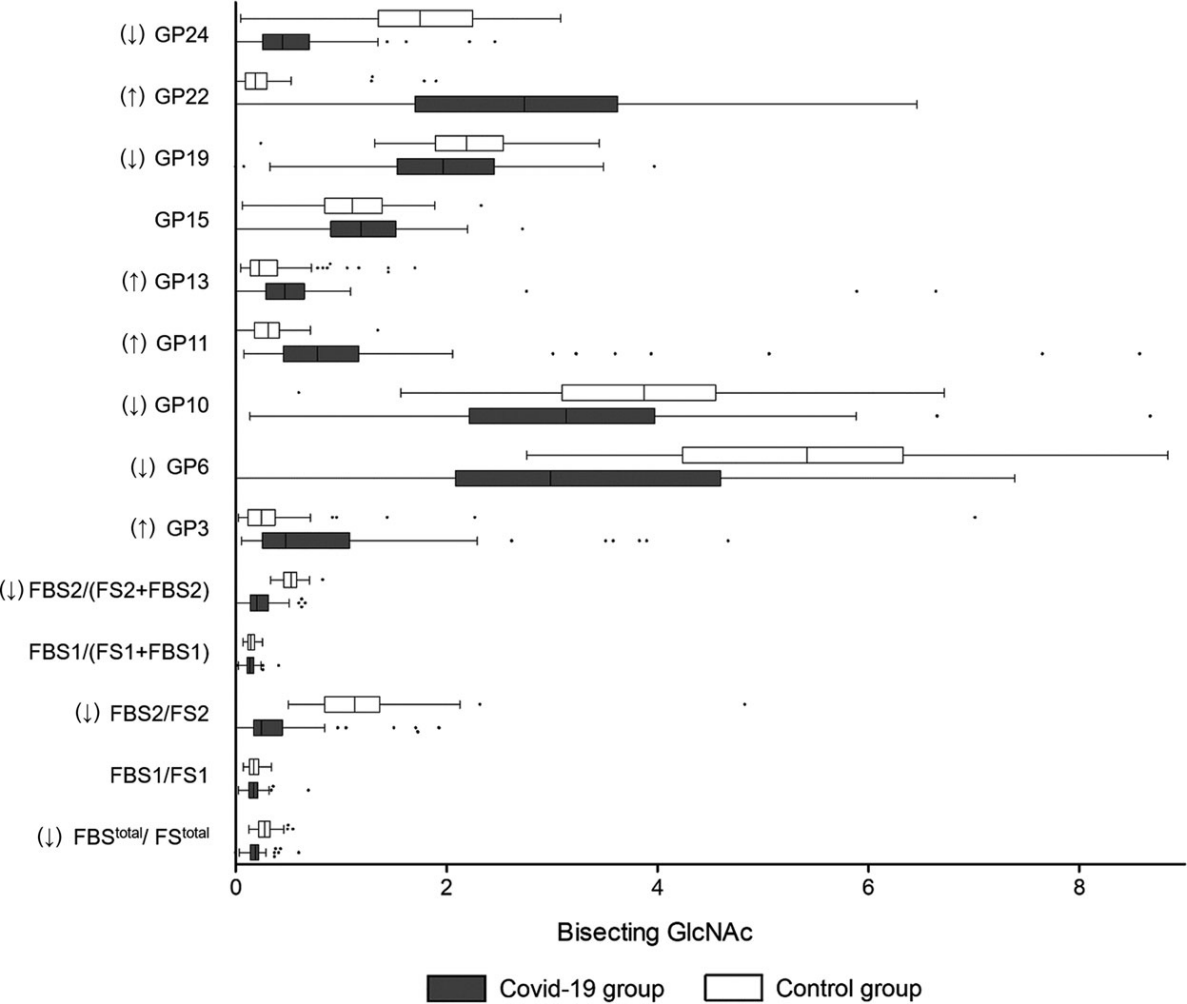
Bisecting GlcNAc on IgG in COVID-19 patients and the healthy controls. B, bisecting N-acetylglucosamine (GlcNAc); F, fucose; G, galactose; GP, glycan peak; S, Sialic acid; ↑, the glycan in COVID-19 group is significantly higher than that in the controls; ↓, the glycan in COVID-19 group is significantly lower than that in the controls.

With regard to bisecting GlcNAc, the decrease of GP6, GP10, GP19, GP24, FBS^total^/FS^total^, FBS2/FS2, and FBS2/(FS2+FBS2) were identified in the COVID-19 cases (**Figure 3**). The percentage of glycans with bisecting GlcNAc was significantly lower in the COVID-19 cases (14.30%) than that in the controls (15.39%) (**Table 2**). Nevertheless, no difference in the total bisecting *N*-GlcNAc was found between the severe and mild cases (13.22% *vs*. 15.38%) (**Table 3**).

### Galactosylation

**Figure 4:**
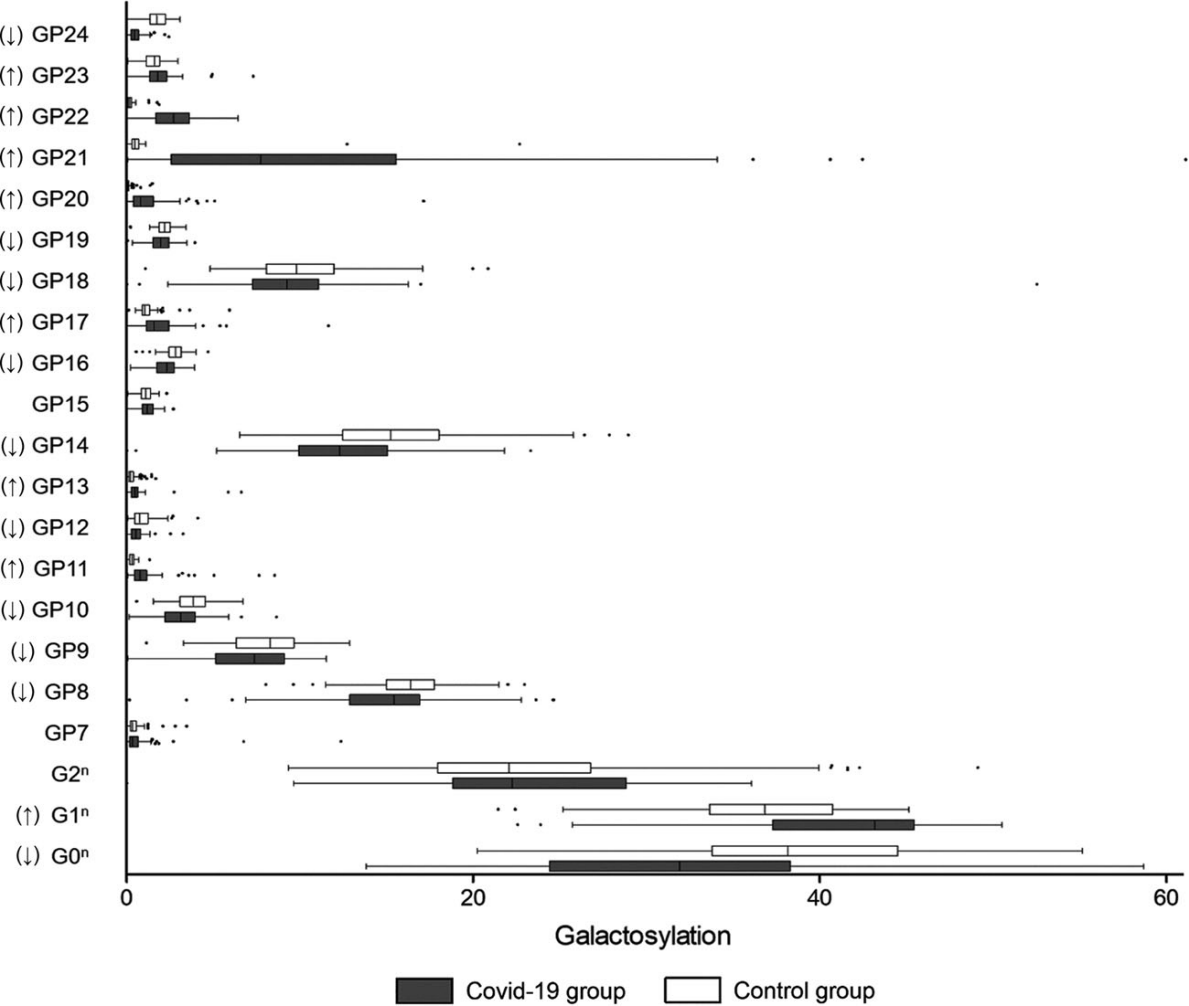
Galactosylation on IgG in COVID-19 patients and the healthy controls. B, bisecting N-acetylglucosamine (GlcNAc); F, fucose; G, galactose; GP, glycan peak; S, Sialic acid; ↑, the glycan in COVID-19 group is significantly higher than that in the controls; ↓, the glycan in COVID-19 group is significantly lower than that in the controls.

Agalactosylated IgG is recognized to be associated with the activation of the lectin-initiated complement pathway in the development of inflammatory diseases (11). As shown in **Figure 4**, the level of galactosylated glycans was significantly higher in the COVID-19 patients (76.13%) when compared to the controls (69.21%) (**Table 2**). Furthermore, a decrease in galactosylated IgG was identified in the severe COVID-19 cases (58.32%) than that in the mild cases (74.07%) (**Table 3**), meaning that agalactosylated IgG plays a role in the inflammatory process among severe COVID-19 cases.

### Time Trend of Immunoglobulin G Glycosylation Profiles

A previous follow-up study demonstrated that the IgG level increases from the first week of symptom onset to the fifth week, then maintains a stable level until the seventh week. In order to explore if the IgG glycosylation profile follows a certain trajectory, we followed-up 23 patients for 4 weeks, and recorded the IgG *N*-glycans in the first week, two to three weeks, and the fourth week after their clinical diagnoses. As shown in **Table S4**, the levels of the 24 initial glycans detected by HILIC-UPLC were not significantly different between the three follow-up durations, indicating that the IgG glycosylation profile is relatively stable after the clinical symptom onset.

### Age- and Sex-Differences in Immunoglobulin G Glycosylation

The relative proportions (%) of the main IgG glycome features were compared between the COVID-19 patients who were divided into three tertile groups. As shown in **Table S5**, there were no significant differences between the three groups. Meanwhile, no differences were observed between men and women (**Table S6**).

## Discussion

**Figure 5:**
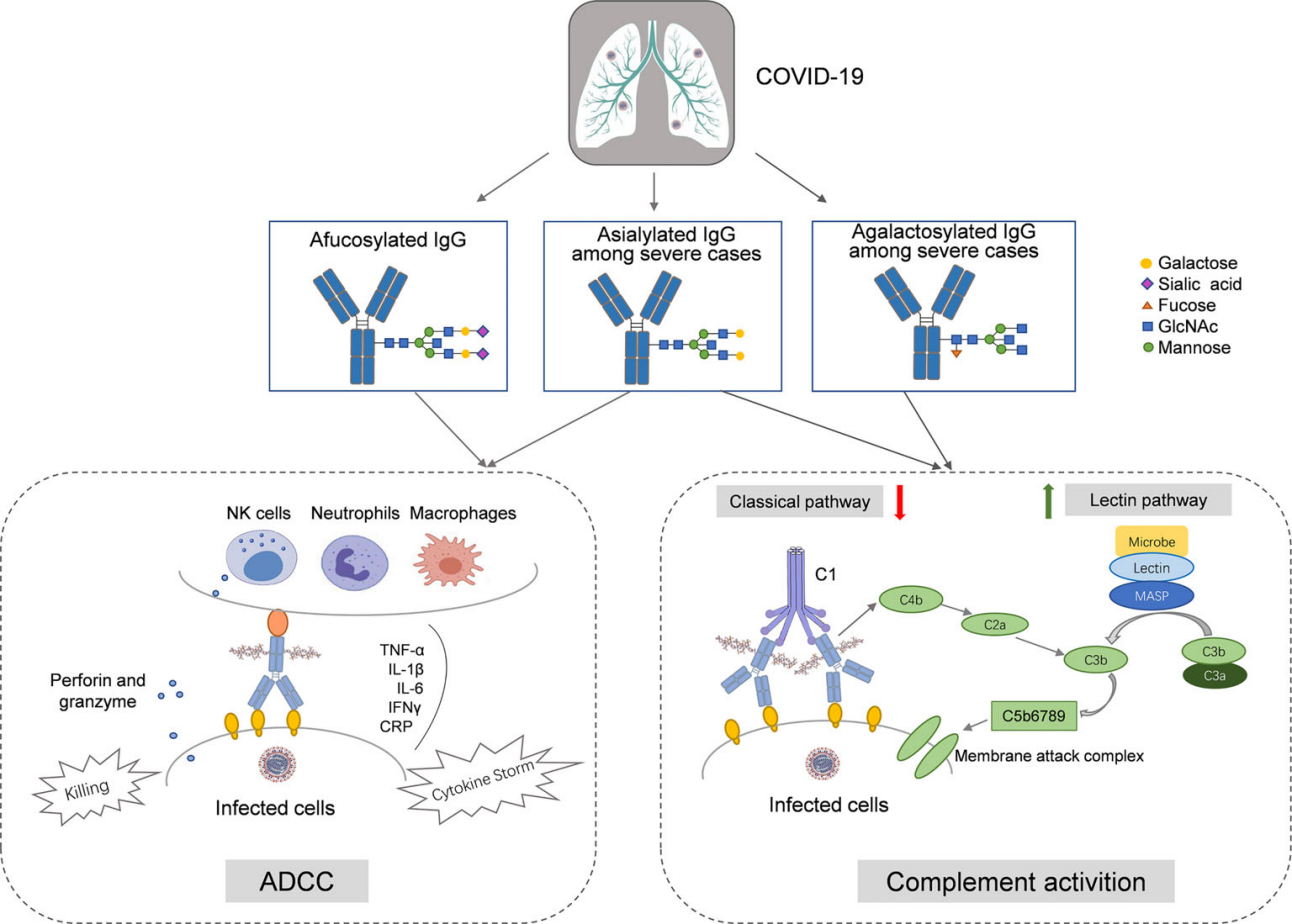
The hypothesized roles of IgG *N*-glycosylation in the immunopathology of COVID-19. In COVID-19 cases, afucosylation of IgG Fc segment activates the antibody-dependent cell mediated cytotoxicity (ADCC) by ligating to FcγRIIIa on natural killer (NK) cells, macrophage, and neutrophil, and then upregulates the release of proinflammatory factors (*e.g.*, IL-1β, IL-6, CRP, TNF-α, and IFN-γ). Among severe cases, agalactosylation and asialylation of IgG upregulates the activation of the lectin-initiated complement pathway, underlying the complex clinical phenotypes during SARS-CoV-2 infection.

Our findings reaffirm that SARS-CoV-2 infection is associated with the absence of the fucosylation of IgG, which triggers ADCC-regulated acute immune responses. Compared to the mild cases, the severe ones might have a lower level of IgG sialylation, which leads to the ADCC-regulated enhancement of inflammatory cytokines and activation of the lectin-initiated alternative complement pathway. In severe cases, the decreases in sialylation and galactosylation might also play a role in the activation of the lectin-initiated alternative complement pathway (**Figure 5**).

Cytokine storm resulting from the overactivation of the innate immune system is one of the key features of severe COVID-19 but significantly varies between individuals (25). Besides, increased levels of pro-inflammatory cytokines are not observed in all severe patients (26). Inter-patient differences in the IgG glycome profiles are apparent and reflect both genetic and environmental determinants (27), which hypothetically contribute to the abnormal expression of glycosyltransferase and glycosidase in plasma cells or B cells, resulting in the different susceptibility of the SARS-CoV-2 infection and severity of the COVID-19. These changes in the IgG glycome may make it possible to switch between anti-inflammatory and pro-inflammatory effects upon antigenic challenge (28). Increased cytokines and chemokines activated peripheral immune cells such as neutrophils and monocytes, penetrate lung tissue, and cause impairments on target cells (29). Consequently, IgG glycosylation might play a cascading role in the progression of COVID-19 pathogenesis.

Here, a significant decrease in the fucosylation of IgG was identified in the COVID-19 cases compared to the healthy controls. This finding is partly consistent with an earlier study (20), in which a decrease in fucosylated IgG against S protein and an increase in fucosylated IgG against N protein were observed among severe COVID-19 patients compared to the mild ones. However, the underlying mechanism that induces these differences was not elucidated. In our study, the total IgGs against multiple antigenic epitopes were examined, including both anti-S and anti-N IgGs. An earlier study showed that COVID-19 patients have substantially higher anti-glycan antibodies (i.e., IgG and IgM) against self-glycans (e.g., *N*-glycans, LacNAc-containing glycans, gangliosides, sialyl Lewis X, and blood group H), compared to healthy controls (30). Nevertheless, it is understood that the absence of fucose increases the capability of IgG to trigger antibody-dependent cell cytotoxicity (ADCC) *via* binding to IgG-specific Fc gamma receptor IIIa (FcγRIIIa) on natural killer (NK) cells, resulting in the enhancement of inflammatory cytokines produced by monocytes, including interleukin-1β (IL-1β), IL-6, tumor necrosis factor-α (TNF-α), C-reactive protein (CRP), and Interferon-γ (IFN-γ) (19, 31). This might be the pathway that IgG afucosylation modulates cytokine storm during the active phase of the SARS-CoV-2 infection.

IgG sialylation-regulated ADCC plays a role in the pro-inflammation and anti-inflammation balance in the pathway similar to fucosylation (32). The absence of sialylation IgG reduces its efficacy to complement-dependent cytotoxicity (CDC) activity *via* C1q binding and leads to an increase in the activation of the lectin-initiated alternative complement pathway (24). In this current study, a higher abundance of sialylated glycans was identified in the COVID-19 group than that in the controls, reflecting the general features in acute immune responses (20). Higher levels of bisecting GlcNAc on IgG enhance ADCC *via* increased FcγRIII binding and elevate the proinflammatory function of IgG (33). We found that the total glycans with bisecting GlcNAc was significantly lower in the COVID-19 cases than that in the controls. This might cause a decrease in the modulation of the inflammatory response among COVID-19 patients, but to a lower degree than that of fucosylation (11). Agalactosylated IgG is associated with the activation of the lectin-initiated complement pathway in the development of inflammatory diseases (34). However, we found that the levels of agalactosylated glycans were decreased in the COVID-19 patients.

The anti-inflammatory and pro-inflammatory balance related to pulmonary immunopathology after SARS-CoV-2 infection might provide a possible explanation for COVID-19 patients with mild *vs.* severe clinical phenotypes. Hereby, we compared the differences of glycan traits between the severe and mild COVID-19 patients to explore the roles of IgG glycosylation in the development of SARS-CoV-2 infection between individuals. Lower levels of sialylated IgG glycans and galactosylated glycans were observed in the severe patients compared to the mild ones. These results support earlier findings in the European COVID-19 patients (18), suggesting that the lectin-initiated alternative complement pathway modulated by the decreased sialylation and galactosylation might induce a severe inflammatory process in severe COVID-19 (11). It was noted that the mild cases (mean age 42.3 years) are significantly younger than the severe cases (mean age 57.0 years). Since aging is one of the main factors inducing the decrease of IgG sialylation and galactosylation (35, 36), the lower levels of sialylation and galactosylation in severe cases might be partly attributed to a higher age.

Only a small number of patients infected with SARS-CoV-2 develop severe COVID-19, suggesting that there are some predisposing factors. Although the pre-existing components of the total IgG pool before SARS-CoV-2 infection are not consistent with that following the infection, it is hypothesized that the glycome profile of IgG might be of little variation, especially in the early stage of infection. Thereby, studies exploring biomarkers that predict disease association are commonly cross-sectional studies, which would not allow us to distinguish whether the reported determinants are pre-existing risk factors (18). Our follow-up study on the IgG glycosylation profiles proved that the IgG glycome profiles are stable from the first week of symptom onset to the fourth week. This makes it predominantly believed that associations between the IgG glycome composition and SARS-CoV-2 infection reflect a pre-existing predisposition. Our finding, in another aspect, validated the previous inference that no correlation exists between any IgG glycosylations and viral RNA load during SARS-CoV-2 virus infection (19).

Despite SARS-CoV-2, two coronavirus-induced epidemics have appeared over the past two decades, including SARS-CoV in 2003 and MERS-CoV in 2012. Researchers have observed an increase in ADCC activity induced by afucosylated anti-MERS antibodies than their fucosylated counterparts, which provides a novel perspective for evaluating the role of the modification of *N*-glycosylation of the Fc segment in improving ADCC activity (37). Moreover, mechanistic experiments demonstrated that during dengue infection, afucosylated IgG promotes FcγRIIIa signaling, and then enhances replication of the virus in monocytes (38). In addition, dengue hemorrhagic fever (DHF) or dengue shock syndrome (DSS) patients respond to infection by producing IgGs with enhanced affinity for the activating FcγRIIIa due to Fc afucosylation (17).

Nearly 45% of individuals infected by SARS-CoV-2 present asymptomatic (39), but there was little opportunity to enroll such participants in our study. No asymptomatic cases were enrolled in our study, which limited the representability of our results. In addition, anti-viral agents and comorbidities might impact the IgG glycome profiles. Despite these limitations, we profiled the IgG glycome in Chinese COVID-19 patients, which contributes to understanding the immune response to the SARS-CoV-2 infection.

In conclusion, compared to the age- and sex-matched healthy controls, COVID-19 patients have a decrease in fucosylated IgG, which upregulates ADCC in acute immune responses. In severe cases, a low level of IgG sialylation contributes to the ADCC-regulated enhancement of inflammatory cytokines. The decreases in sialylation and galactosylation that activate the lectin-initiated alternative complement pathway also play a role in COVID-19 pathogenesis, which underlines the complex clinical phenotypes of the SARS-CoV-2 infection.

## Data Availability Statement

The raw data supporting the conclusions of this article will be made available by the authors, without undue reservation.

## Ethics Statement

The studies involving human participants were reviewed and approved by Chongqing General Hospital Research Ethics Committee (No. S2020-021-01). The patients/participants provided their written informed consent to participate in this study.

## Author Contributions

WW and PL conceived the research and initiated the project. HH, PCL, HY, and MW designed the experiments. WW and PL supervised the overall project design and execution. CH, YL, PCL, MZ, JW, and YX participated in data analysis and interpretation. YW, QM, and DL helped collect data and analyze the structure. HH, PCL, and HY wrote the manuscript. All authors contributed to the article and approved the submitted version.

## Funding

This work was supported by the National Natural Science Foundation of China (81773527, 81973138), the China-Australia International Collaborative Grant (NHMRC APP1112767, NSFC 81561128020) and the European Commission Horizon 2020 Framework Programme (PRODEMOS-779238).

## Conflict of Interest

Author QM is employed by Bioyong Technologics.

The remaining authors declare that the research was conducted in the absence of any commercial or financial relationships that could be construed as a potential conflict of interest.

## Publisher’s Note

All claims expressed in this article are solely those of the authors and do not necessarily represent those of their affiliated organizations, or those of the publisher, the editors and the reviewers. Any product that may be evaluated in this article, or claim that may be made by its manufacturer, is not guaranteed or endorsed by the publisher.
