# Supplementary material for: Profile of Immunoglobulin G N-Glycome in COVID-19 Patients: A Case-Control Study
Source: Front Immunol. 2021 Sep 23;12:748566. doi: 10.3389/fimmu.2021.748566 (PMC8495247; doi:10.3389/fimmu.2021.748566)
Supplement: Supplementary file 1 [file Table_1.docx]

**[****Supplementary Material]**

**Table S1** Comparison of initial glycans between COVID-19 patients and healthy controls

| Glycans | COVID-19 patients  (n=104) | Controls  (n=104) | *Z* | *P* |
| --- | --- | --- | --- | --- |
| GP1 | 0.27 (0.13-0.85) | 0.15 (0.08-0.24) | 4.216 | <0.001 |
| GP2 | 0.43 (0.23-1.02) | 0.66 (0.40-0.94) | 2.340 | 0.019 |
| GP3 | 0.48 (0.25-1.08) | 0.25 (0.12-0.38) | 5.714 | <0.001 |
| GP4 | 16.47 (10.86-23.20) | 24.21 (19.94-29.33) | 5.808 | <0.001 |
| GP5 | 0.49 (0.3-0.83) | 0.08 (0.03-0.18) | 10.505 | <0.001 |
| GP6 | 2.99 (2.09-4.60) | 5.42 (4.24-6.33) | 8.346 | <0.001 |
| GP7 | 0.34 (0.18-0.68) | 0.34 (0.23-0.56) | 0.126 | 0.900 |
| GP8 | 15.44 (12.89-16.92) | 16.41 (14.99-17.76) | 3.030 | 0.002 |
| GP9 | 7.37 (5.15-9.13) | 8.28 (6.33-9.67) | 2.303 | 0.021 |
| GP10 | 3.14 (2.22-3.97) | 3.88 (3.10-4.55) | 4.190 | <0.001 |
| GP11 | 0.78 (0.45-1.17) | 0.31 (0.18-0.42) | 8.685 | <0.001 |
| GP12 | 0.54 (0.29-0.80) | 0.76 (0.47-1.26) | 4.074 | <0.001 |
| GP13 | 0.47 (0.29-0.66) | 0.23 (0.14-0.40) | 5.457 | <0.001 |
| GP14 | 12.31 (9.94-15.07) | 15.25 (12.48-18.03) | 4.837 | <0.001 |
| GP15 | 1.19 (0.90-1.52) | 1.11 (0.85-1.39) | 1.565 | 0.118 |
| GP16 | 2.33 (1.74-2.76) | 2.86 (2.47-3.18) | 5.925 | <0.001 |
| GP17 | 1.59 (1.13-2.45) | 1.05 (0.92-1.32) | 5.607 | <0.001 |
| GP18 | 9.27 (7.27-11.09) | 9.81 (8.07-11.99) | 2.157 | 0.031 |
| GP19 | 1.97 (1.53-2.45) | 2.19 (1.89-2.54) | 2.996 | 0.003 |
| GP20 | 0.80 (0.39-1.57) | 0.07 (0.03-0.12) | 10.130 | <0.001 |
| GP21 | 7.74 (2.60-15.54) | 0.48 (0.32-0.71) | 10.955 | <0.001 |
| GP22 | 2.74 (1.70-3.63) | 0.19 (0.09-0.30) | 11.272 | <0.001 |
| GP23 | 1.82 (1.34-2.34) | 1.62 (1.14-1.93) | 2.346 | 0.019 |
| GP24 | 0.45 (0.26-0.70) | 1.75 (1.36-2.25) | 11.077 | <0.001 |

GP, glycan peak

**Table S2** Comparison of derived glycans between COVID-19 patients and healthy controls

| Derived traits of glycosylation | COVID-19 patients  (n=104) | Controls  (n=104) | *Z* | *P* | |
| --- | --- | --- | --- | --- | --- |
| FGS/(FG+FGS) | 28.30 (24.30-31.39) | 26.58 (23.79-29.35) | 2.078 | 0.038 |  |
| FBGS / (FBG+FBGS) | 32.13 (26.99-40.36) | 43.62 (37.12-49.65) | 7.254 | <0.001 |  |
| FGS / (F+FG+FGS) | 21.02 (16.77-25.00) | 18.17 (15.30-21.38) | 3.000 | 0.003 |  |
| FBGS / (FB+FBG+FBGS) | 22.83 (17.77-29.73) | 28.15 (23.44-31.79) | 3.885 | <0.001 |  |
| FG1S1 / (FG1+FG1S1) | 9.27 (7.22-11.78) | 10.30 (8.99-11.96) | 2.673 | 0.008 |  |
| FG2S1 / (FG2+FG2S1+FG2S2) | 39.20 (34.77-41.73) | 36.96 (35.18-39.59) | 3.074 | 0.002 |  |
| FG2S2 / (FG2+FG2S1+FG2S2) | 7.92 (5.10-9.60) | 5.86 (4.45-7.45) | 4.037 | <0.001 |  |
| FBG2S1 / (FBG2+FBG2S1+FBG2S2) | 53.16 (45.53-57.50) | 43.42 (39.85-47.70) | 6.166 | <0.001 |  |
| FBG2S2 / (FBG2+FBG2S1+FBG2S2) | 12.17 (8.18-19.64) | 35.05 (30.23-39.44) | 10.695 | <0.001 |  |
| FtotalS1 / FtotalS2 | 5.83 (4.72-7.54) | 4.43 (3.78-5.86) | 5.138 | <0.001 |  |
| FS1 / FS2 | 6.31 (5.25-9.38) | 8.21 (6.54-10.76) | 3.570 | <0.001 |  |
| FBS1 / FBS2 | 4.55 (2.76-6.57) | 1.27 (1.05-1.54) | 10.108 | <0.001 |  |
| FBStotal / FStotal | 0.19 (0.15-0.22) | 0.28 (0.22-0.33) | 7.496 | <0.001 |  |
| FBS1 / FS1 | 0.17 (0.13-0.21) | 0.17 (0.13-0.22) | 0.710 | 0.478 |  |
| FBS1 / (FS1+FBS1) | 0.14 (0.11-0.17) | 0.15 (0.12-0.18) | 0.770 | 0.441 |  |
| FBS2 / FS2 | 0.25 (0.17-0.45) | 1.13 (0.85-1.37) | 11.092 | <0.001 |  |
| FBS2 / (FS2+FBS2) | 0.20 (0.14-0.31) | 0.53 (0.46-0.58) | 11.090 | <0.001 |  |
| GP1n | 0.39 (0.18-1.16) | 0.18 (0.10-0.31) | 5.368 | <0.001 |  |
| GP2n | 0.77 (0.37-1.25) | 0.85 (0.49-1.11) | 0.882 | 0.378 |  |
| GP4n | 24.74 (19.08-30.75) | 30.77 (26.46-36.01) | 4.702 | <0.001 |  |
| GP5n | 0.76 (0.43-1.39) | 0.09 (0.04-0.22) | 10.771 | <0.001 |  |
| GP6n | 4.80 (3.58-6.25) | 6.80 (5.72-7.77) | 7.341 | <0.001 |  |
| GP7n | 0.56 (0.26-0.96) | 0.44 (0.28-0.72) | 1.659 | 0.097 |  |
| GP8n | 23.16 (20.28-24.51) | 20.68 (19.32-22.31) | 4.405 | <0.001 |  |
| GP9n | 11.53 (8.51-13.23) | 10.49 (8.18-12.18) | 1.949 | 0.051 |  |
| GP10n | 4.88 (3.66-5.89) | 4.90 (3.96-5.64) | 0.016 | 0.987 |  |
| GP11n | 1.28 (0.63-1.83) | 0.41 (0.22-0.52) | 9.119 | <0.001 |  |
| GP12n | 0.75 (0.45-1.29) | 1.00 (0.57-1.59) | 2.134 | 0.033 |  |
| GP13n | 0.77 (0.43-0.96) | 0.28 (0.18-0.55) | 6.543 | <0.001 |  |
| GP14n | 18.83 (15.54-24.29) | 19.47 (15.00-23.75) | 0.447 | 0.655 |  |
| GP15n | 1.79 (1.40-2.57) | 1.39 (1.07-1.74) | 4.509 | <0.001 |  |
| G0n | 31.92 (24.41-28.31) | 38.17 (33.79-44.51) | 5.101 | <0.001 |  |
| G1n | 43.16 (37.32-45.44) | 36.82 (33.64-40.74) | 5.986 | <0.001 |  |
| G2n | 22.27 (18.83-28.83) | 22.06 (17.95-26.77) | 0.659 | 0.510 |  |
| Fn total | 95.15 (92.48-96.08) | 96.85 (95.49-97.66) | 6.437 | <0.001 |  |
| FG0n total / G0n | 97.59 (96.57-98.68) | 97.90 (96.71-98.64) | 0.611 | 0.541 |  |
| FG1n total / G1n | 98.67 (97.42-99.26) | 98.81 (98.22-99.22) | 1.036 | 0.300 |  |
| FG2n total / G2n | 90.12 (86.22-92.69) | 92.87 (90.40-94.45) | 4.343 | <0.001 |  |
| Fn | 81.74 (77.81-83.92) | 83.07 (81.26-84.93) | 3.110 | 0.002 |  |
| FG0n / G0n | 82.70 (78.90-85.15) | 80.34 (77.29-82.60) | 3.122 | 0.002 |  |
| FG1n / G1n | 83.45 (79.11-85.83) | 84.27 (82.11-86.31) | 2.207 | 0.027 |  |
| FG2n / G2n | 84.48 (80.22-87.13) | 87.49 (85.27-89.44) | 4.656 | <0.001 |  |
| FBn | 13.18 (11.26-15.37) | 13.46 (11.43-15.62) | 0.781 | 0.435 |  |
| FBG0n / G0n | 15.37 (12.35-19.01) | 17.26 (15.15-19.66) | 3.721 | <0.001 |  |
| FBG1n / G1n | 15.28 (12.28-18.65) | 14.04 (12.26-16.42) | 1.622 | 0.105 |  |
| FBG2n / G2n | 8.14 (6.32-9.61) | 6.45 (5.10-7.68) | 5.329 | <0.001 |  |
| FBn / Fn | 15.89 (13.42-1.29) | 16.18 (13.66-18.97) | 0.161 | 0.872 |  |
| FBn / Fn total | 13.71 (11.83-16.17) | 13.93 (12.01-15.95) | 0.157 | 0.876 |  |
| Fn / (Bn + FBn) | 5.83 (4.74-6.95) | 6.11 (5.15-6.88) | 1.029 | 0.304 |  |
| Bn / (Fn + FBn) | 8.03 (4.54-10.23) | 2.92 (1.86-5.62) | 6.596 | <0.001 |  |
| FBG2n / FG2n | 0.10 (0.07-0.12) | 0.07 (0.06-0.09) | 5.780 | <0.001 |  |
| FBG2n / (FG2n + FBG2n) | 8.82 (6.90-1071) | 6.84 (5.27-8.17) | 5.664 | <0.001 |  |
| FG2n / (BG2n + FBG2n) | 7.43 (5.80-9.17) | 10.77 (9.20-14.24) | 7.576 | <0.001 |  |
| BG2n / (FG2n + FBG2n) | 32.93 (22.92-51.08) | 16.30 (8.99-23.99) | 7.041 | <0.001 |  |

F, fucose; S, Sialic acid; B, bisecting N-acetylglucosamine (GlcNAc); G, galactose

**Table S3** Comparison of core fucosylation in severe and mild COVID-19 patients

| Core fucosylation | Severe patients  (n=18) | Mild patients  (n=86) | *Z* | *P* | |
| --- | --- | --- | --- | --- | --- |
| Fn total | 94.54 (92.08-95.75) | 95.04 (92.77-96.44) | 0.516 | 0.606 |  |
| FG0n total / G0n | 97.39 (96.55-97.68) | 97.55 (94.57-98.85) | 1.246 | 0.213 |  |
| FG1n total / G1n | 98.47 (97.55-99.20) | 99.20 (97.51-99.52) | 0.026 | 0.979 |  |
| FG2n total / G2n | 90.41 (86.35-92.20) | 91.18 (87.46-94.62) | 0.034 | 0.973 |  |
| Fn | 81.29 (79.99-84.01) | 83.10 (78.93-84.06) | 0.481 | 0.630 |  |
| FG0n / G0n | 84.13 (81.40-86.47) | 81.59 (78.62-83.94) | 1.856 | 0.063 |  |
| FG1n / G1n | 84.06 (81.44-87.26) | 83.53 (80.74-86.89) | 1.169 | 0.243 |  |
| FG2n / G2n | 83.86 (79.87-86.81) | 86.05 (80.39-89.50) | 0.292 | 0.770 |  |

F, fucose; G, galactose

**Table S4** Comparison of initial glycans of COVID-19 patients in different follow-up durations

| Glycans | Week 1  (n=23) | Weeks 2-3  (n=23) | Week 4  (n=23) | *χ^2^* | *P* |
| --- | --- | --- | --- | --- | --- |
| GP1 | 0.28 (0.09-0.87) | 0.19 (0.08-0.53) | 0.21 (0.14-0.69) | 0.733 | 0.693 |
| GP2 | 0.48 (0.19-0.99) | 0.31 (0.14-1.25) | 0.39 (0.23-0.81) | 0.406 | 0.816 |
| GP3 | 0.51 (0.28-1.23) | 0.40 (0.16-1.65) | 0.50 (0.29-0.77) | 0.498 | 0.780 |
| GP4 | 15.39 (11.02-18.66) | 14.90 (10.48-20.34) | 15.16 (9.06-19.06) | 0.197 | 0.906 |
| GP5 | 0.46 (0.34-0.96) | 0.53 (0.12-0.94) | 0.54 (0.36-0.81) | 0.388 | 0.824 |
| GP6 | 3.17 (2.55-4.47) | 2.87 (1.68-4.72) | 2.81 (2.46-3.14) | 1.632 | 0.442 |
| GP7 | 0.50 (0.23-0.77) | 0.44 (0.20-1.07) | 0.37 (0.16-0.55) | 3.335 | 0.189 |
| GP8 | 15.84 (12.93-16.97) | 15.12 (10.70-18.14) | 14.14 (11.86-16.90) | 0.564 | 0.754 |
| GP9 | 8.12 (5.78-8.98) | 6.97 (5.42-9.06) | 8.12 (6.12-9.86) | 0.817 | 0.665 |
| GP10 | 3.43 (2.46-4.19) | 3.07 (2.31-4.11) | 3.16 (1.81-3.58) | 3.241 | 0.198 |
| GP11 | 0.72 (0.42-1.09) | 0.85 (0.53-1.25) | 0.76 (0.43-1.11) | 0.375 | 0.829 |
| GP12 | 0.71 (0.37-0.97) | 0.54 (0.34-0.80) | 0.60 (0.31-0.88) | 0.714 | 0.700 |
| GP13 | 0.59 (0.26-0.70) | 0.51 (0.23-0.71) | 0.54 (0.36-0.68) | 0.665 | 0.717 |
| GP14 | 11.99 (9.90-15.07) | 12.31 (10.50-15.76) | 12.31 (8.41-14.06) | 0.457 | 0.796 |
| GP15 | 1.16 (0.90-1.67) | 1.19 (1.09-1.52) | 1.23 (0.91-1.56) | 0.025 | 0.987 |
| GP16 | 2.35 (1.67-2.81) | 2.07 (1.82-2.83) | 2.44 (1.91-2.62) | 0.061 | 0.970 |
| GP17 | 1.58 (1.15-2.06) | 1.95 (1.23-3.09) | 2.28 (1.46-3.27) | 2.937 | 0.230 |
| GP18 | 8.93 (6.65-10.62) | 9.67 (7.98-11.37) | 8.54 (7.54-10.00) | 1.235 | 0.539 |
| GP19 | 1.85 (1.40-2.35) | 2.08 (1.69-2.29) | 2.09 (1.67-2.57) | 1.861 | 0.394 |
| GP20 | 0.98 (0.36-1.76) | 0.75 (0.31-1.82) | 1.02 (0.68-2.69) | 1.933 | 0.380 |
| GP21 | 7.93 (5.85-15.89) | 9.45 (3.25-17.29) | 9.51 (3.71-23.14) | 0.642 | 0.725 |
| GP22 | 2.67 (1.77-3.55) | 2.82 (1.75-3.90) | 3.18 (2.36-3.91) | 2.662 | 0.264 |
| GP23 | 1.93 (1.40-2.38) | 1.97 (1.28-2.33) | 1.83 (1.60-2.41) | 0.179 | 0.914 |
| GP24 | 0.45 (0.31-0.64) | 0.47 (0.28-0.87) | 0.45 (0.22-0.92) | 0.380 | 0.827 |

GP, glycan peak

**Table S5** Difference in main IgG glycome features of COVID-19 patients between age groups

| Summary glycans | Tertile 1  (n=35) | Tertile 2  (n=34) | Tertile 3  (n=35) | *Z* | *P* |
| --- | --- | --- | --- | --- | --- |
| Fucosylation (%) | 83.23 (71.81-90.20) | 82.60 (73.62-87.03) | 80.97 (70.40-86.99) | 0.445 | 0.801 |
| Bisecting GlcNAc (%) | 13.50 (11.97-15.43) | 14.49 (13.16-15.85) | 14.57 (13.40-15.49) | 1.800 | 0.407 |
| Galactosylation (%) | 78.61 (68.39-83.97) | 75.51 (71.60-78.51) | 75.91 (60.31-84.35) | 1.291 | 0.524 |
| Sialylation (%) | 28.97 (17.28-39.36) | 27.81 (23.64-35.19) | 32.21 (20.32-42.97) | 0.732 | 0.694 |

GlcNAc, N-acetylglucosamine

**Table S6** Difference in main IgG glycome features of COVID-19 patients between sex groups

| Summary glycans | Men  (n=50) | Women  (n=54) | *Z* | *P* |
| --- | --- | --- | --- | --- |
| Fucosylation (%) | 80.97 (71.11-86.49) | 84.24 (79.00-90.22) | 1.422 | 0.155 |
| Bisecting GlcNAc (%) | 13.93 (12.32-15.50) | 14.89 (13.50-16.38) | 1.256 | 0.209 |
| Galactosylation (%) | 75.63 (62.88-81.13) | 76.12 (67.88-82.95) | 0.865 | 0.387 |
| Sialylation (%) | 28.17 (22.37-39.57) | 30.24 (19.48-36.55) | 0.644 | 0.520 |

GlcNAc, N-acetylglucosamine
